# Deregulated enhancer‐promoter communication in cancer through altered nuclear architecture

**DOI:** 10.1002/ijc.35424

**Published:** 2025-04-12

**Authors:** Isabelle Seufert, Claire Vargas, Sina Jasmin Wille, Karsten Rippe

**Affiliations:** ^1^ German Cancer Research Center (DKFZ) Heidelberg Division of Chromatin Networks Heidelberg Germany; ^2^ Center for Quantitative Analysis of Molecular and Cellular Biosystems (BioQuant) Heidelberg University Heidelberg Germany

**Keywords:** enhancer hijacking, extrachromosomal DNA, fusion protein, onco‐condensates, phase separation, transcription regulation

## Abstract

Enhancers are critical regulators of gene expression. Structural variations in cancer genomes can lead to enhancer hijacking, where oncogenes are activated by mistargeted enhancer activity. Novel enhancer‐promoter interactions may also arise through chromosomal rearrangements that create extrachromosomal DNA elements. Additionally, fusion proteins and other mutation‐induced alterations in protein properties can lead to the aberrant assembly of proteins into large complexes on the size scale of 0.1–1 μm termed onco‐condensates. Transcription factors and co‐activators accumulate with cis‐regulatory elements in these structures, driving oncogenic programs. Here, we review current evidence of how altered genome architecture and macromolecular assembly result in deregulated enhancer‐promoter communication. We discuss emerging strategies to exploit these mechanisms for clinical applications.

## Graphical abstract

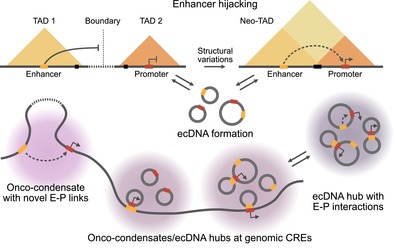

**Abbreviations**
ATAC‐seq: assay for transposase‐accessible chromatin using sequencing
BRD4: bromodomain‐containing protein 4
ChIP‐seq: chromatin immunoprecipitation sequencing
CRE: cis‐regulatory element
CTCF: CCCTC‐binding factor
ecDNA: extrachromosomal DNA
ENL: eleven‐nineteen leukemia
E‐P: enhancer‐promoter
EWSR1: Ewing sarcoma RNA‐binding protein 1
FLI1: friend leukemia integration 1
GFI1: growth factor independence 1
H3K27ac/H3K27me3: histone H3 lysine 27 acetylation/trimethylation
H3K4me1/H3K4me3: histone H3 lysine 4 mono‐/trimethylation
H3K9ac/me3: histone H3 lysine 9 acetylation/trimethylation
HOXA9: homeobox A9
HPV: human papillomavirus
IDR: intrinsically disordered region
MED1: mediator of RNA polymerase II transcription subunit 1
NUP98: nucleoporin 98
NUT: nuclear protein in testis
RNAP II: RNA polymerase II
SMC: structural maintenance of chromosomes
SWI/SNF: Switch/Sucrose Non‐Fermentable
TAD: topologically associating domain
TAG: transcription factor activity gradient
TF: transcription factor
TSS: transcription start site
YY1: Yin Yang 1

## 1 INTRODUCTION

Precise control of gene expression depends on cis‐regulatory elements (CREs)—evolutionarily conserved, non‐coding genomic regions typically ranging from 100 to 1000 base pairs in size and containing transcription factor (TF) binding sites.^1^ Through interactions with their target transcription start site (TSS), CREs orchestrate cell type‐specific gene expression programs during development and in response to environmental signals. Disruption of CRE function, particularly through altered enhancer activity, is increasingly recognized as a key mechanism driving cancer development.^2^, ^3^

Based on their genomic location and function, CREs are classified as promoters when they are proximal to their target TSS^4^ or as enhancers, silencers, and boundary elements when distal to their targets.^5^ Enhancers are known for regulating genes from distances that can exceed 1 Mb.^1^ While traditionally viewed as distinct elements, promoters and enhancers share many features.^6^, ^7^ Both produce RNA transcripts, share chromatin signatures, and can swap functions, with promoters enhancing distant gene transcription.^8^, ^9^, ^10^ At the same time, it is estimated that the human genome encodes between 0.1 and 1 million CREs^11^, ^12^, ^13^—a number significantly higher than the approximately 20,000 protein‐coding genes, which points to complex regulatory mechanisms by distal CREs.

High‐throughput reporter assays, such as STARR‐seq, have identified sequence motifs and regulatory elements critical for effective enhancer‐promoter (E‐P) interactions.^14^ Notably, many enhancer sequences show specificity for particular promoters, even when brought into contact through chromosomal rearrangements. This underscores the importance of sequence compatibility in shaping functional interactions.^15^, ^16^ Accordingly, understanding E‐P communication requires deciphering how these elements interact with their target promoters. This process includes local CRE activity influenced by their chromatin state, the three‐dimensional organization of chromatin that facilitates long‐range interactions, and the assembly of TFs and co‐activators into dynamic supramolecular structures, which concentrate and coordinate regulatory interactions.

### 1.1 CRE chromatin states

**FIGURE 1:**
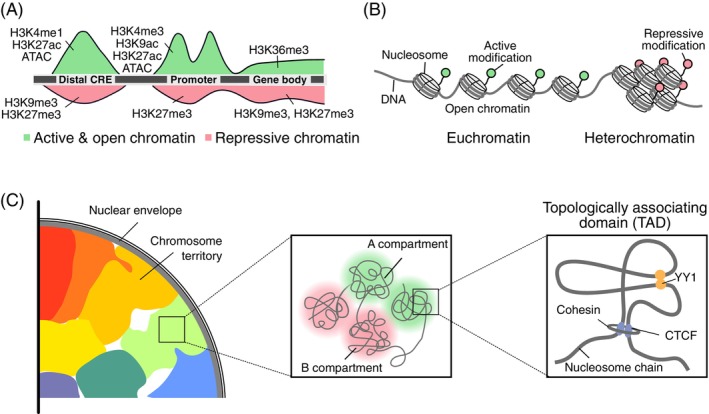
Chromatin state, organization, and three‐dimensional architecture. (A) Epigenetic histone post‐translational modifications and chromatin accessibility as measured by chromatin immunoprecipitation sequencing (ChIP‐seq) and the assay for transposase‐accessible chromatin using sequencing (ATAC‐seq), respectively, determine the local chromatin state. Activating (green, high accessibility) and repressive (red, low accessibility) states are shown for promoter, gene body, and distal a distal CRE. (B) Local organization of the chromatin fiber into euchromatic and heterochromatic regions. (C) The nuclear chromatin is organized into chromosome territories and A/B compartments. Cohesin and CCCTC‐binding factor (CTCF)‐mediated loops between distal chromatin sites establish topologically associating domains (TADs). Figure adapted from Ref. 17. H3K4me1/H3K4me3, histone H3 lysine 4 mono‐/trimethylation; H3K9ac/me3, histone H3 lysine 9 acetylation/trimethylation; H3K27ac/me3, histone H3 lysine 27 acetylation/trimethylation; H3K36me3, histone H3 lysine 36 trimethylation.

E‐P communication occurs in the context of local chromatin states at CREs, which reflect and influence regulatory activity (Figure 1A,B). Active enhancers and promoters are marked by specific DNA sequence features, accessible chromatin, unique histone variants (such as H2A.Z and H3.3), and activating histone modifications—particularly histone H3 lysine 27 acetylation (H3K27ac) and histone H3 lysine 4 mono‐/trimethylation (H3K4me1/H3K4me3).^18^, ^19^, ^20^, ^21^, ^22^, ^23^ These modifications aid in recruiting transcriptional machinery and maintaining active regulatory hubs. In contrast, repressive modifications such as histone H3 lysine 27 trimethylation (H3K27me3) and histone H3 lysine 9 trimethylation (H3K9me3), along with DNA methylation, can impede enhancer activity.^24^ Importantly, CRE chromatin states are dynamically regulated by the writers, readers, and erasers of these epigenetic modifications, enabling cells to adjust their activity in response to developmental or environmental signals.^25^

### 1.2 Chromatin topology‐mediated E‐P communication

In addition to the local CRE state, the three‐dimensional, higher‐order chromatin organization is a crucial factor in E‐P communication.^26^, ^27^ During interphase, chromosomes occupy distinct territories within the nucleus (Figure 1C).^28^ Within these chromosome territories, chromatin is organized into so‐called A and B compartments.^29^ The A compartments are euchromatic, generally active, and typically located in the nuclear interior, while B compartments primarily consist of inactive chromatin and are found near the nuclear envelope.^29^ In the euchromatic A compartments, distal chromatin regions can interact through targeted chromatin loop formation or random interactions along the dynamic chromatin chain.^26^, ^28^, ^30^, ^31^ CCCTC‐binding factor (CTCF) and cohesin create targeted structural loops between specific chromatin regions.^32^, ^33^ These loops increase the likelihood of dynamic spatial contacts within the intervening chromatin, forming stochastically interacting regions known as topologically associating domains (TADs).^34^, ^35^ Additionally, the mediator complex and specific TFs, such as Yin Yang 1 (YY1) or NANOG, can facilitate targeted chromatin contacts.^36^, ^37^, ^38^ Together, targeted and random contacts shape higher‐order chromatin organization, which regulates transcription by bringing multiple distal CREs into close spatial proximity.^26^, ^27^, ^30^

### 1.3 Transcription factor assembly and E‐P communication

Recent studies underscore the significance of spatial nuclear organization in transcriptional regulation, where transcription‐associated factors assemble into distinct nuclear structures known as transcription factories, hubs, or condensates.^39^, ^40^, ^41^, ^42^, ^43^, ^44^ These structures, which range from 50 nm to 1 μm in size, can form through multivalent interactions that frequently involve intrinsically disordered regions (IDRs) present in TFs and co‐activators, playing a vital role in transcription regulation and genome organization.^45^, ^46^, ^47^ A key function of IDRs is to facilitate interactions that drive the assembly of transcription compartments, enriching RNA polymerase II (RNAP II) and its co‐activators, such as Mediator of RNAP II transcription subunit 1 (MED1) and Bromodomain‐containing protein 4 (BRD4) with CREs. In cancer, the aberrant formation and composition of these nuclear subcompartments can result in pathological gene expression patterns, particularly the upregulation of oncogene expression. Therefore, the deregulation of such transcriptional assemblies represents a critical mechanism through which E‐P communication becomes altered in cancer.

### 1.4 Deregulated E‐P communication in cancer

**FIGURE 2:**
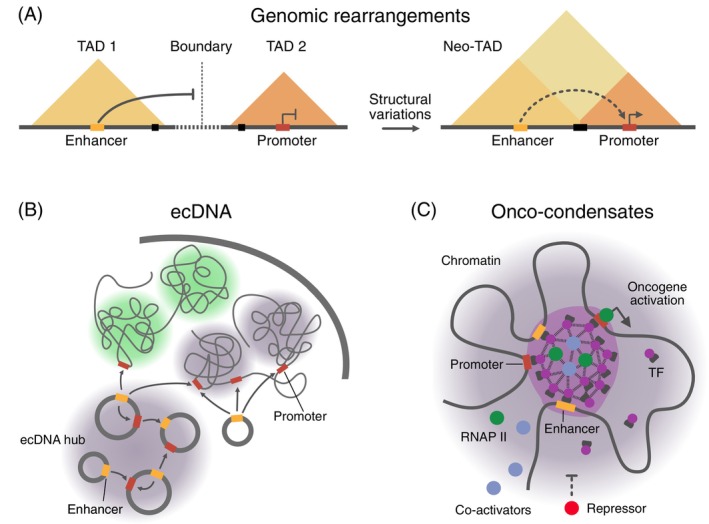
Mechanisms of enhancer deregulation via altering nuclear architecture. (A) Genomic rearrangements. Structural variation in the genome can change the genome and 3D chromatin organization so that enhancers would target another promoter, referred to as “enhancer hijacking.” (B) Enhancer‐promoter (E‐P) communication on extrachromosomal DNA (ecDNA). Novel E‐P links could be generated on ecDNA elements. In addition, ecDNA promoters could act in trans with enhancers in the genome and/or enhancer sequences on ecDNA could act as mobile enhancer elements that act on other genes in trans. (C) Onco‐condensates. Aberrant assemblies of transcription factor, co‐activators, and RNAP II, for example driven by fusion proteins, could assemble together with cis‐regulatory elements into supramolecular complexes with altered gene regulation activity. RNAP II, RNA polymerase II; TAD, topologically associating domains; TF, transcription factor.

It is well‐established that changes between repressive and active chromatin states at enhancers and promoters are a hallmark of deregulated gene expression in cancer.^3^, ^48^, ^49^ These changes could, for instance, arise directly from mutations within the CREs or the regulators of the epigenome. The Switch/Sucrose non‐fermentable (SWI/SNF) chromatin remodeler, which is mutated in approximately 20% of cancers,^50^ is a critical factor for local enhancer activity. It is directed to CREs by histone modifications and is essential for opening chromatin at enhancers for TF binding.^51^ In addition, other mechanisms can mistarget enhancer activity in a complex manner, which is the focus of the present review. First, structural variations in cancer genomes can rewire E‐P interactions. This “enhancer hijacking” can place oncogenes under the control of ectopic enhancers within the genome (Figure 2A).^52^, ^53^, ^54^, ^91^ Second, the formation of extrachromosomal DNA elements (ecDNA) can concentrate, amplify, or create oncogenic E‐P interactions (Figure 2B).^55^, ^56^, ^58^, ^59^, ^60^, ^61^, ^62^ In addition, the aberrant assembly of TFs on the 0.1–1 μm scale into “oncogenic condensates” or “onco‐condensates” could drive tumorigenesis via multiple pathways that have direct links to enhancer activity (Figure 2C).^63^, ^64^, ^65^

In summary, deregulated E‐P communication in cancer arises from a complex interplay of various factors beyond direct changes to the local chromatin state and activity of enhancers. Here, we discuss how aberrant genome architecture and protein assembly at CREs can lead to deregulated enhancer activity. We also highlight emerging therapeutic opportunities that target these deregulated enhancers in cancer. Understanding the principles of E‐P communication and its disruption in cancer will be crucial for identifying new biomarkers and treatment strategies.

## 2 ENHANCER HIJACKING THROUGH ALTERED GENOME SEQUENCE

### 2.1 Mechanisms of E‐P communication at a distance

**FIGURE 3:**
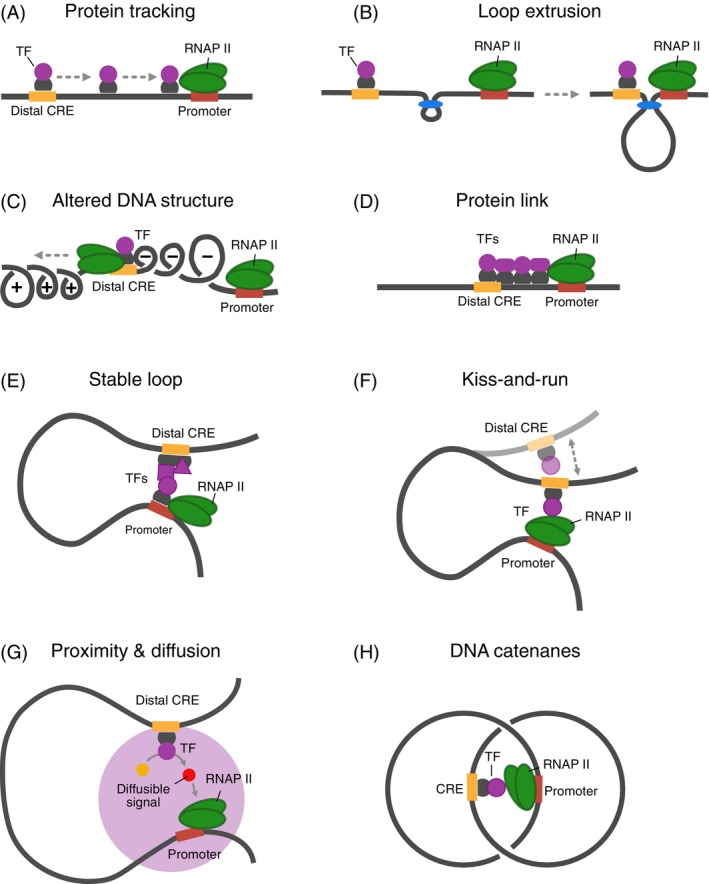
Models for DNA‐mediated enhancer‐promoter (E‐P) communication. (A) Protein tracking model. An activator binds to a distal cis‐regulatory elements (CRE) and tracks along the DNA until it reaches its target promoter. (B) Loop extrusion model. Protein‐mediated active loop extrusion brings a distal CRE close to the promoter. (C) DNA structure model. An altered DNA conformation is transmitted from the distal CRE to the promoter. (D) Protein link model. Various transcription factors (TFs) oligomerize between the distal CRE and promoter. (E) Stable loop model. A chromatin loop establishes stable spatial contact between the distal CRE and promoter. (F) Kiss‐and‐run model. Transient E‐P contacts mediate the interaction between TFs at the distal CRE and proteins at the promoter. (G) Proximity and diffusion model. The distal CRE and promoter are in spatial proximity but do not contact each other. Promoter activation happens through the diffusion of a signaling protein generated at the distal CRE, which then translocates to the target promoter. (H) DNA catenanes. It has been demonstrated that enhancer interactions with promoters can activate expression in trans within DNA catenanes. Figure adapted from Ref. 17. RNAP II, RNA polymerase II.

Most current studies of E‐P communication focus on interactions mediated by chromatin looping. However, in many instances, data are lacking to evaluate if alternative mechanisms are involved in deregulating this process in cancer (Figure 3).^66^, ^67^, ^68^, ^69^, ^70^ Accordingly, there is some uncertainty about the molecular determinants of enhancer activity in normal cells and how it is mistargeted in cancer cells.^68^, ^71^, ^72^ In the *protein tracking* model (Figure 3A), communication is mediated by a protein that binds to a specific site on the DNA and actively moves along the DNA strand to its target activation site. This mechanism has been demonstrated for the late promoter of bacteriophage T4.^73^ *Loop extrusion* (Figure 3B) is an energy‐consuming protein translocation relative to the DNA that can be driven by the structural maintenance of chromosome (SMC) protein complexes.^74^, ^75^ Additionally, it has been proposed that transcription factories reel in the DNA instead of having RNAP II track along it.^39^ These protein‐driven movements of DNA could bring enhancers and promoters closer together.

The *transmission of an altered DNA structure* between the enhancer and the promoter could also activate transcription (Figure 3C). For instance, transcription at the enhancer could create a locally unwound region of DNA in front of and behind the polymerase.^76^, ^77^ Since partial unwinding facilitates the melting of the promoter DNA and transcription initiation, transcription of one gene can stimulate transcription of a second gene located upstream. Such coupling between two promoters has been described for the leu‐500 promoter in *Escherichia coli*.^78^ Furthermore, studies have shown that DNA supercoiling can enhance the activation rate of a prokaryotic enhancer.^79^, ^80^ Transcription‐induced supercoiling may also significantly affect E‐P communication and transcription regulation in eukaryotes.^81^, ^82^

The *protein link* model suggests that TFs oligomerize between enhancers and promoters to create a functional bridge (not necessarily through chromatin‐bound factors) that transmits the activation signal from the enhancer to the promoter (Figure 3D). This protein link is established at upstream elements or sequences that exhibit enhancer activity near their target TSS.^83^, ^84^ If the intervening DNA is looped out, this interaction transforms into a *stable chromatin loop* through a complex of TFs and co‐factors at a distal CRE and the transcription machinery at the promoter (Figure 3E). Alternatively, in the *kiss‐and‐run* model (Figure 3F), only a transient interaction is necessary for activation. Finally, in the *proximity and diffusion* model, the two CREs are spatially close (typically 200–300 nm) but do not directly contact each other (Figure 3G). In this case, activation could occur via diffusion of a TF post‐translationally modified at the enhancer to the promoter as proposed for the TF activity gradient (TAG) model.^68^ The diffusive TF translocations could occur through the nucleoplasm or within a liquid‐like protein droplet between the interacting CREs.

It is noted that E‐P communication via chromatin looping models (Figure 3E–G) differs from the models depicted in Figure 3A–D because a direct DNA connection between the two sites on the DNA is not required for their function. This has been experimentally tested with *DNA catenanes* (Figure 3H), where promoter and enhancer are located on two separate circular DNA molecules.^85^, ^86^ They are kept in close spatial proximity due to the topological interlinking of the two circles. Thus, although endogenous enhancers, by definition, are associated with activation in cis, mobile isolated enhancer elements could also regulate transcription in trans of a target gene in cis. This is functionally relevant for activation by ecDNA, as discussed below.

### 2.2 Structural variations driving enhancer hijacking

TADs, TAD substructures, and the higher‐order assembly of TADs into an A/B compartment structure (Figure 1C) typically constrain E‐P interactions.^27^, ^87^, ^88^ TAD boundaries can be maintained through convergent CTCF binding sites and cohesin‐mediated loop extrusion, creating insulated neighborhoods that restrict E‐P interactions. However, cancer genomes frequently harbor structural variations that can create new E‐P pairs.^3^, ^53^, ^89^, ^90^ These include chromosomal translocations, inversions, and deletions that bring previously distant regulatory elements into proximity or bypass normal regulatory boundaries (Figure 2A). Through this enhancer hijacking, strong enhancers can activate oncogenes due to altered genome architecture.

Chromosomal translocations currently represent the most frequent and well‐documented mechanisms of enhancer hijacking in cancer.^91^ These events can juxtapose strong enhancers near proto‐oncogenes, as first described for the IgH enhancer driving *MYC* expression in Burkitt's lymphoma.^54^ Recent studies have identified numerous translocation‐mediated enhancer hijacking events across various cancer types.^92^, ^93^, ^94^ However, enhancer hijacking can also occur through other structural variations, including inversions,^52^ focal amplifications,^95^ and the formation of ecDNA, as discussed below.

One key mechanism involves the loss of boundary insulation through mutations, deletions, or epigenetic alterations of CTCF binding sites. A prominent example occurs in gliomas with isocitrate dehydrogenase (IDH) mutations, where hypermethylation of CTCF binding sites weakens TAD boundaries, allowing the *PDGFRA* oncogene to interact with and become activated by enhancers from neighboring TADs.^96^ Structural variations in cancer genomes can also create new TAD structures, known as neo‐TADs, with aberrant regulatory interactions. Examples include medulloblastoma, where genomic rearrangements lead to the activation of growth factor independence 1 (GFI1) family oncogenes,^52^ neuroblastoma, where neo‐TADs result in *MYC* activation,^95^ leukemia involving the activation of *EVI1*/*MECOM*,^94^, ^97^ and salivary gland tumors, where these changes drive *NR4A3* expression.^98^ However, it is important to note that not all TAD disruptions lead to altered gene expression. An analysis of over 2500 cancer genomes revealed that only approximately 14% of TAD disruptions resulted in changes in gene expression.^99^

These findings suggest that additional factors, such as enhancer strength, cellular context, and the compatibility of the newly created E‐P interactions, influence the functional impact of TAD alterations. Thus, enhancer hijacking involves a complex interplay between topological and chromatin state changes. One example of this link is the emergence of H3K4me3 domains spreading several kilobases in size at oncogene promoters observed in multiple myeloma following the relocation of enhancers near oncogenes like *CCND1*.^100^ The same study noted similar cancer‐specific broad H3K4me3 domains associated with super‐enhancer hijacking of other common oncogenes in B‐cell and T‐cell malignancies. The formation of broad H3K4me3 domains appears to be facilitated by increased E‐P contacts and the recruitment of H3K4 methyltransferases, creating a self‐reinforcing regulatory hub that sustains high oncogene expression.^101^ These findings underscore how structural variations can alter both enhancer targeting and the promoter chromatin state to drive oncogenic transcription.

## 3 NOVEL ENHANCER‐PROMOTER INTERACTIONS MEDIATED BY EXTRACHROMOSOMAL DNA

**FIGURE 4:**
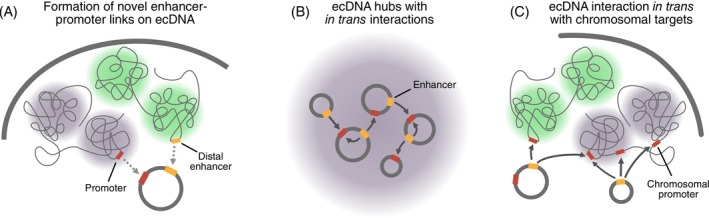
Mechanisms of extrachromosomal DNA (ecDNA)‐mediated enhancer‐promoter communication. (A) Formation of a novel ecDNA harboring oncogenes and hijacked enhancers. (B) Various types of ecDNAs assemble in ecDNA hubs that facilitate in trans interactions between enhancers and oncogenes located on different ecDNAs. (C) Interaction of enhancers on ecDNA in trans with chromosomal oncogenes.

Formation of ecDNA can drive deregulated E‐P communication through both intra‐ and intermolecular interactions.^55^, ^56^, ^58^, ^59^, ^60^, ^61^, ^62^, ^89^ Generally, ecDNAs are characterized by a decondensed state with high chromatin accessibility, which facilitates TF binding and E‐P interactions.^55^ Because they lack centromeres, ecDNAs segregate unevenly during cell division, resulting in high copy numbers of ecDNAs in a subset of cells. Recent studies have identified several mechanisms through which ecDNAs can drive oncogene expression (Figure 4).^58^, ^59^, ^60^, ^61^, ^102^ In ecDNAs, novel E‐P interactions can be created by combining oncogenes with enhancers from different chromosomal locations, as shown for ERBB2 and MYC enhancers (Figure 4A).^89^ Related hybrid ecDNAs have been identified in human papillomavirus (HPV)‐related oropharyngeal cancer.^56^ These elements produce fusion transcripts that combine HPV promoters and oncogenes with downstream human sequences. Within the same ecDNA, enhancers can be co‐amplified with oncogenes, increasing enhancer‐oncogene interactions.^57^ Importantly, ecDNAs can also form that exclusively harbor enhancers, co‐existing alongside those containing oncogenes and oncogene‐enhancer amplicons (Figure 4B). By assembling into ecDNA hubs, enhancer interactions can occur in trans between ecDNAs, resulting in high oncogene expression (Figure 4B).^58^ However, enhancers on ecDNAs can act not only in trans with other ecDNAs but also on genes located on the chromosomes (Figure 4C).^59^

One example that encompasses several aspects described above is *MYCN* amplification on ecDNAs in neuroblastoma: (i) Local enhancers can be replaced by enhancers hijacked from more distal regions of chromosome 2.^60^ (ii) ecDNAs have been reported to form hubs.^58^ (iii) Hi‐C data reveal in trans interactions between different ecDNA amplicons in neuroblastoma cell lines.^58^ These mechanisms result in elevated *MYCN* transcription, leading to the over‐expression of the protein N‐MYC. These findings underscore the diverse and dynamic ways that ecDNA contributes to oncogene activation, emphasizing its critical role in cancer pathogenesis and its potential as a therapeutic target.

## 4 DEREGULATED ENHANCER ACTIVITY THROUGH THE FORMATION OF ONCO‐CONDENSATES

Recent studies have reported that the aberrant assembly of proteins and RNA into onco‐condensates may be crucial to tumorigenesis.^63^, ^64^, ^65^, ^103^, ^104^, ^105^ Here, we adopt this terminology. However, it is emphasized that we use the term onco‐condensate solely for abnormal macromolecular protein/RNA assemblies in cancer cells, without any implications about their formation mechanisms (i.e., whether they arise from phase separation or other processes) or their functions (i.e., whether they contribute to tumorigenesis or occur downstream of this process). Onco‐condensates frequently involve TFs and thus can affect transcription (thus overlapping with transcriptional condensates), locally concentrate proteins from the transcriptional machinery, and drive the expression of cancer‐promoting genes. They may operate through other mechanisms, such as sequestering tumor suppressors or altering signal transduction pathways.

### 4.1 Organization and properties of transcription factor assemblies

**FIGURE 5:**
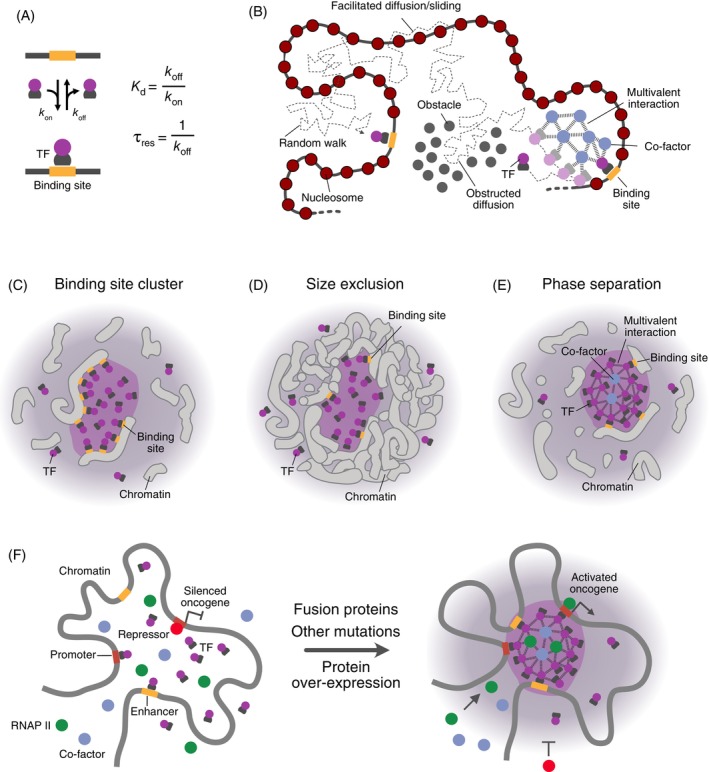
Principles of transcription factor interactions with chromatin. (A) TF‐binding to a specific site in the genome. TFs bind and dissociate with rates *k*_on_ and *k*_off_ that determine *K*_d_ as well as the residence time *τ*_res_ in the bound state. (B) Model of TF movement through the nucleus. (C) Local TF enrichment through binding to a cluster of binding sites. (D) Local TF enrichment due to size exclusion from densely packed surrounding chromatin. (E) Local TF enrichment by phase separation into liquid droplets. (F) The transcriptional effect of TF accumulation. Acquisition of an intrinsically disordered region, conformational changes, and an increase in TF concentration heighten multivalent interactions between transcription‐associated proteins, leading to the formation of aberrant transcription hubs through phase separation or other processes. These assemblies mediate new enhancer‐promoter interactions that, in turn, activate oncogenic pathways. Figure adapted from Ref. 17.

TFs and co‐activators are generally found at low concentrations in the nucleus and contain different functional domains: DNA‐binding domains that recognize specific motifs and effector domains that regulate transcription.^106^, ^107^ They bind to their target sites with specific kinetic on (*k*_on_) and off (*k*_off_) rates that determine the equilibrium dissociation constant *K*_d_. The residence time *τ*_res_ in the bound state is derived from 1/*k*_off_, typically ranging from 1 to 10 s (Figure 5A). TFs diffuse in a random walk through the nucleus (Figure 5B). This diffusion can be facilitated along the chromatin fiber or by molecular crowding. Multivalent interactions via IDRs may guide this diffusive search process. The local TF concentration must be sufficiently high to ensure high occupancy of the binding sites, which may involve different mechanisms^108^, ^109^: (i) Local clustering of multiple binding sites could enhance TF concentration through simultaneous binding (Figure 5C).^110^ (ii) Size exclusion from densely packed chromatin may restrict TFs to specific, less occupied regions (Figure 5B,D).^111^ (iii) Physicochemical phase separation driven by multivalent IDR interactions may lead to the formation of phase‐separated liquid droplets or other assemblies, particularly at super‐enhancers (Figure 5E).^41^, ^112^

The role of phase separation in assembling endogenous transcription hubs and/or increasing transcription activity remains controversial. Often, the assembly of phase‐separated transcriptional condensates depends on protein over‐expression, and it is unclear whether it would take place under endogenous conditions. Moreover, several studies report no activation or even repressive effects on gene expression when the TF concentration is elevated to enhance TF assembly.^107^, ^113^, ^114^, ^115^

### 4.2 Onco‐condensate mediated transcription activation

TFs must efficiently locate their binding motifs within large eukaryotic genomes.^116^, ^117^ During this process, TFs navigate through the nucleus by diffusion in a “random walk” (Figure 5B, left). This movement may be aided by diffusion along the one‐dimensional chromatin fiber or through macromolecular crowding (Figure 5B, middle). Additionally, IDRs can create nonspecific multivalent interactions with other locally enriched chromatin factors, potentially directing TFs toward specific DNA motifs (Figure 5B, right).^118^, ^119^ Consequently, onco‐condensates can drive transcriptional activation through several mechanisms: (i) Concentrating the transcriptional machinery by recruiting and concentrating RNAP II, TFs, and co‐activators while excluding transcriptional repressors may enhance transcriptional activity.^43^, ^65^ (ii) The confinement and local increase in TF concentration could reduce the search time for binding sites and increase occupancy (Figure 5B).^120^, ^121^ (iii) Facilitating the formation of new E‐P interactions (Figure 5F).^122^ (iv) Inducing specific epigenetic modification patterns that establish active or repressive chromatin states at CREs.^3^

### 4.3 Formation mechanisms of onco‐condensates

In cancer cells, the aberrant accumulation of TFs on chromatin into onco‐condensates may arise from various processes (Figure 5F). Well‐established drivers are genomic rearrangements like chromosomal translocations that create fusion proteins.^63^, ^123^, ^124^, ^125^ It is estimated that ~16.5% of cancer cases are driven by fusion proteins,^126^ with exceptionally high frequencies in childhood cancers like leukemias and translocation‐related sarcomas (20% of sarcoma cases). Most nuclear fusion proteins combine an IDR from one protein with a chromatin‐binding domain from another protein, typically a TF. The IDRs can establish nonspecific multivalent interactions with other locally enriched chromatin factors.^118^, ^119^ This architecture enables the formation of onco‐condensates through phase separation or other mechanisms, affecting both transcription and 3D genome organization.^122^, ^127^, ^128^ Examples are the EWSR1‐FLI1 fusion in Ewing Sarcoma, which combines the IDR of Ewing Sarcoma RNA‐binding protein 1 (EWSR1) with the TF friend leukemia integration 1 (FLI1)^127^, ^129^, ^130^ and the NUP98‐HOXA9 fusion of nucleoporin 98 (NUP98) and the homeobox A9 (HOXA9) TF as well as other homeodomain proteins.^122^, ^131^, ^132^ The TFs gain enhanced interaction capabilities in these fusions compared to their wild‐type counterparts, redefining the binding sites, biophysical properties, and interaction partners.

Mutations that modify protein conformation to promote higher‐order assembly are another driver of the formation of onco‐condensates. For instance, hotspot mutations in SHP2 disrupt intramolecular interactions, exposing domains that mediate multivalent electrostatic interactions and condensate formation.^65^, ^133^ Additionally, small insertions or deletions, such as the three‐amino‐acid insertion in the mutated ENL‐T1 of the transcription regulator eleven‐nineteen leukemia (ENL), can induce onco‐condensate formation and drive tumorigenesis.^134^, ^135^ Finally, the over‐expression of oncoproteins due to deregulation or amplification can lead to condensate formation when protein levels surpass the critical concentration necessary for phase separation.^65^, ^120^, ^121^, ^136^ Recent studies have provided detailed examples of these mechanisms in cancer. The over‐expression of the proto‐oncogene *MYCN*, through the mechanisms described above (Figure 5F), leads to aberrant transcriptionally active onco‐condensates that activate oncogenic pathways while inhibiting tumor suppressors.^115^

## 5 INTEGRATION OF STRUCTURAL AND MOLECULAR MECHANISMS OF ENHANCER DEREGULATION

From the discussion above about E‐P communications, it is clear that an integrated perspective on structural changes, chromatin topology, and onco‐condensates is essential for a better understanding of how these processes affect deregulated E‐P communication in cancer.

### 5.1 Mechanistic relationships between genome structure and onco‐condensates

Cancer‐specific chromosomal rearrangements can simultaneously influence 3D genome organization and condensate formation, raising critical questions about their mechanistic relationship. A prime example is the NUP98‐HOXA9 fusion protein in acute myeloid leukemia, which creates onco‐condensates while promoting CTCF‐independent chromatin loops that establish new E‐P interactions.^122^ Similarly, the EWSR1‐FLI1 fusion generates nuclear onco‐condensates in Ewing sarcoma. Both in vitro and cellular studies demonstrated that EWSR1‐FLI1 relocalizes to microsatellite repeats, functioning as a distal enhancer by creating onco‐condensates and activating oncogenic pathways.^105^, ^127^ This relocalization reshapes local chromatin architecture, driving oncogenic transcription programs.^65^, ^137^ The dual activity of these fusion proteins suggests complex connections between their local accumulation and genome organization. The IDRs that drive protein assembly may also enable new chromatin interactions by creating local environments that concentrate factors necessary for loop formation.^45^, ^46^, ^47^ Furthermore, structural variations that cause enhancer hijacking could create new microenvironments that promote condensate formation by increasing local concentrations of regulatory factors.

### 5.2 Temporal dynamics and causality

A crucial question in understanding enhancer deregulation revolves around the temporal order and causal relationships between structural and molecular changes.^88^ While enhancer hijacking and onco‐condensate formation are linked to cancer, their temporal sequence remains poorly understood. Recent live‐cell imaging studies indicate that many E‐P interactions may be more dynamic than previously thought, with interaction times ranging from seconds to minutes instead of forming stable loops.^31^, ^33^, ^138^, ^139^, ^140^, ^141^

The dynamic nature raises several questions about what is cause and what is consequence. Do changes in 3D genome organization create conditions that promote onco‐condensate formation, or do onco‐condensates help establish and maintain new chromatin interactions? Evidence from the NUP98‐HOXA9 system suggests that onco‐condensates may initiate new E‐P interactions, which subsequently become stabilized through additional mechanisms.^122^ However, the general applicability of this model has yet to be established across different cancer contexts.

### 5.3 Specificity determinants

A critical challenge in enhancer regulation is achieving specificity in target gene activation. This challenge is particularly relevant for understanding how this specificity is altered in cancer and leads to oncogenic gene expression profiles. Given that a typical human nucleus of 100 µm^3^ in volume contains 200 genes per μm^3^, it is crucial to understand how specificity is achieved through the combined action of genome architecture and protein assembly. Several factors contribute to enhancer specificity. At the structural level, pre‐existing genome architecture, including TAD boundaries and other architectural features, can limit which E‐P interactions are possible.^3^, ^88^ Additional specificity could arise from the selective recruitment of factors into onco‐condensates. Alternating blocks of oppositely charged amino acids within IDRs create a molecular selectivity system. These charge patterns can simultaneously facilitate the recruitment of positive regulators while excluding negative transcription regulators.^132^, ^142^, ^143^ This molecular selectivity could work in conjunction with structural constraints to ensure appropriate target gene activation. Examining these specificity mechanisms in cancer is key to predicting which enhancer alterations drive tumorigenesis. This analysis may reveal how cancer cells sustain oncogenic transcription and whether enhancer deregulation broadly affects transcription or only a few critical targets.

### 5.4 Cross‐talk between enhancer deregulation mechanisms

**FIGURE 6:**
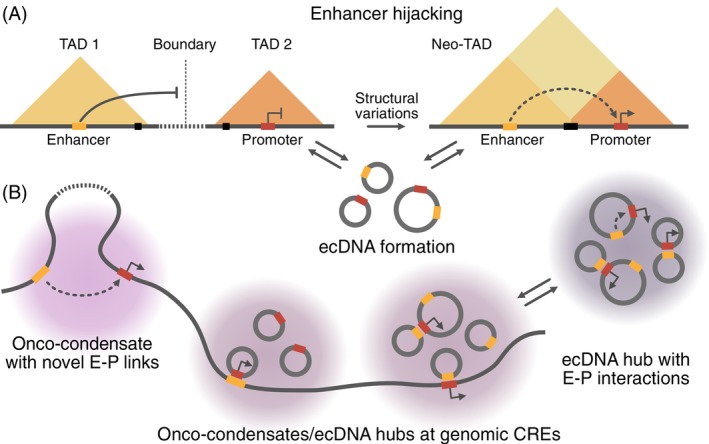
Integration of enhancer deregulation mechanisms. (A) Enhancer hijacking occurs when structural variations disrupt topologically associating domains (TAD) boundaries, creating a neo‐TAD with novel regulatory interactions. These structural variations may also lead to the formation of extrachromosomal DNA (ecDNA) that contains both enhancer and promoter elements. (B) Onco‐condensates can promote new enhancer‐promoter (E‐P) interactions by bringing spatially separated genomic regions closer together (left side). At the same time, ecDNAs have been shown to form hubs where enhancers can activate promoters through in trans interactions (right side). Accordingly, ecDNAs could also be enriched within onco‐condensates, generating novel microenvironments for E‐P communication.

Enhancer deregulation mechanisms drive oncogenic transcription within interconnected networks. Chromothripsis and other complex rearrangements can not only lead to enhancer hijacking but may also generate ecDNAs through the excision and circularization of CREs, creating novel E‐P combinations (Figure 6A).^89^, ^144^ Similarly, both onco‐condensates and ecDNA hubs could facilitate novel E‐P interactions by bringing spatially separated promoters and enhancers into proximity (Figure 6B).^58^, ^122^ These processes may occur independently or allow ecDNA‐mediated trans‐regulatory networks to assemble within onco‐condensates when driven by compatible multivalent interactions (Figure 6B). Such a mechanism aligns with the finding that ecDNAs can form chromatin connectivity hubs, acting as super‐enhancers and creating hotspots for aberrant transcription.^59^ The cross‐talk between different mechanisms of E‐P communication likely involves changes in epigenetic patterns as well. Deregulated chromatin modifiers create permissive environments around hijacked enhancers, as discussed above in relation to the formation of broad H3K4me3 domains at oncogene promoters.^100^ These broad domains may serve as nucleation sites where CRE‐associated proteins could accumulate and assemble into onco‐condensates to drive oncogenic gene expression programs.^145^

## 6 IMPLICATIONS FOR CLINICAL APPLICATIONS

Understanding integrated mechanisms of aberrant E‐P communication creates novel opportunities for developing targeted cancer therapies. Current approaches primarily focus on epigenetic drugs that modify active or repressive CRE states,^146^ but insights into nuclear architecture‐mediated enhancer deregulation can reveal additional therapeutic pathways.^65^ One promising strategy aims to restore proper insulation or block inappropriate E‐P interactions, as reviewed previously.^3^, ^147^ For instance, targeting BRD4‐nuclear protein in testis (NUT) fusion proteins in NUT carcinoma disrupts oncogenic enhancer domains,^148^ effectively blocking aberrant oncogene expression.

Onco‐condensates are emerging as a new class of drug targets that could influence the action of anti‐cancer drugs by directing them to specific genomic regions.^65^, ^149^, ^150^, ^151^, ^152^, ^153^ The compound ET516 has demonstrated the potential to disrupt androgen receptor condensates in castration‐resistant prostate cancer,^154^ while bis‐ANS can affect the phase separation of certain IDR‐containing proteins.^65^, ^155^ Combined therapeutic approaches targeting compensatory structural and molecular mechanisms may be the most effective for durable therapeutic responses. These could simultaneously affect phase separation and architectural proteins or exploit synthetic lethal interactions.^71^

Several key challenges remain for the successful clinical translation of E‐P communication research in cancer: (i) Developing improved multi‐omics methods and combinatorial biomarkers that link structural variations to mistargeted enhancers and detect the activity of ecDNAs and onco‐condensates. (ii) Expanding studies of onco‐condensate formation from cell lines to primary tumors to validate their clinical relevance. (iii) Advancing drugs that modify the activity of ecDNA hubs and onco‐condensate properties. (iv) Selectively targeting cancer‐specific CRE deregulation, which will require strategies that distinguish between physiological and pathological E‐P communication. (v) Addressing the functional redundancy of enhancer networks that involve multiple parallel oncogenic E‐P interactions.^58^, ^59^

## 7 CONCLUSIONS

This review highlights how enhancer hijacking, ecDNAs, and onco‐condensates conspire to drive aberrant E‐P interactions. Understanding their interplay and how enhancers activate transcription from 200 to 300 nm distances without direct contact^68^, ^88^ will be crucial for targeting E‐P communication in cancer therapy. Mapping long‐range chromatin contacts along with regions of locally increased TF activity^156^ or connecting spatial transcriptomics with 3D genome and chromatin organization^157^, ^158^ could illuminate the underlying mechanisms. Gaining deeper insights into these regulatory processes will connect fundamental science with clinical applications by uncovering how distal CREs drive oncogenic transcription. Although significant challenges remain, this emerging view on transcriptional deregulation has the potential to transform cancer diagnostics and therapy by focusing on the previously underappreciated role of E‐P communication.

## AUTHOR CONTRIBUTIONS

**Isabelle Seufert:** Writing – original draft; writing – review and editing. **Claire Vargas:** Writing – review and editing. **Sina Jasmin Wille:** Writing – review and editing. **Karsten Rippe:** Writing – original draft; writing – review and editing; conceptualization.

## FUNDING INFORMATION

This work was supported by German Research Foundation (DFG) grants RI 1283/17‐1 and RI 1283/15‐2 within Priority Program SPP2202 and Research Group FOR2674, respectively.

## CONFLICT OF INTEREST STATEMENT

All authors declare that they have no conflict of interest.
